# Are autonomous cities our urban future?

**DOI:** 10.1038/s41467-018-04505-0

**Published:** 2018-05-29

**Authors:** Barbara Norman

**Affiliations:** Canberra Urban and Regional Futures (CURF), University of Canberra, ACT Climate Change Council, Canberra, ACT Australia

## Abstract

Cities are rapidly expanding in size, wealth and power, with some now larger than nation states. Smart city solutions and strong global urban networks are developing to manage massive urban growth. However, cities exist within a wider system and it may take more than technological advances, innovation and city autonomy to develop a sustainable urban future.

As the world moves towards a global population of nearly 10 billion by 2050, most of which will be urban, the pressures on our cities already facing challenges of urban health, climate change, social justice and urban governance, are likely to increase. The challenge is almost overwhelming and will require innovative policy solutions way beyond ‘business as usual’^1^.

A range of urban policy solutions have recently been pursued, including national urban strategies for better cities, city deals between national and local governments and most recently the concept of smart cities—‘the effective integration of physical, digital and human systems in the built environment to deliver a sustainable, prosperous and inclusive future for its citizens’^2^. Furthermore, the involvement of national governments in urban policy varies significantly between countries^3^, as does the consistency between city and national government agendas, at times at odds, for example, over environmental goals.

The future development of mega urban regions, a number of which are already larger than some nation states, will wield significant wealth and power, and with that more responsibility to develop and implement their own futures (e.g., Hong Kong-Shenhzen-Guangzhou, Nagoya-Osaka-Kyoto-Kobe, Rio de Janeiro-São Paulo)^4^. The necessary investment in infrastructure alone to cater for urban growth will be enormous, estimated to grow globally from $4 trillion per year in 2012 to more than $9 trillion per year by 2025 (ref. ^5^).

As cities grow they will individually achieve greater wealth and power in decision-making and influence with their reach extended further as they coalescence into global networks. The C40 global cities network (http://www.c40.org/) is an example where capital cities are collaborating on major challenges and sharing knowledge and experience on critical issues such as renewable energy, urban health and well-being. Singapore is a good example of where benefits have been reaped as a result of a clear vision to become more sustainable (economically, environmentally and socially) through innovative urban design and development based on sustainable principles. For example, Singapore has managed to increase its urban density and ‘greenery’ at the same time, and stands as a leading example to other growing cities (ref. ^6^ and see pages 133–134 in ref. ^7^). Yet for all their wealth and power, cities remain dependent in the wider context. Singapore, for example, is highly reliant on Malaysia for water resources.

## Smart cities

The concept of ‘autonomous cities’ is not new in the sense that informal settlements have existed for millennia, and many independent indigenous communities live in remote locations around the world. However, the concept is remerging as cities increasingly set their own agendas. An example is the United States where, in contrast to national policy, sub-national governments with a wide range of non-government and private sector partners have created an alliance of action on climate change: We Are Still In^8^. Action taken includes green infrastructure, smart climate design and the building of more resilient neighbourhoods for the impacts of climate change (heat, sea level rise and extreme events).

Many of the above urban challenges were at the centre of discussions at the recent World Urban Forum (WUF)—held every 2 years and hosted by UN Habitat to advance implementation of the recently adopted UN New Urban Agenda (Quito 2016). WUF 9 displayed the groundswell of urban innovation occurring across the globe and showcased the embrace of new technology including localised renewable energy, smart and green infrastructure, integrated public transit and e-democracy designed to improve community input to local city plans. There is no doubt that the urban data ecosystem is expanding with digital mapping, smart asset management and urban mobility and that these developments have the capacity to rapidly improve urban management^9^.

A key driver for smarter cities is planning for the impacts of climate change and the expected increase in urban heat island effects and extreme events (droughts, floods and coastal storms). In this context, the policy of smart cities has the potential to make a major contribution. The inaugural IPCC conference on Cities and Climate Change came to a similar conclusion, as stated by Debra Roberts, Co-Chair of IPCC Working Group II 'Business-as-usual will not save the world. This conference disrupted the traditional story of the world’s cities to show how science can partner with policy and practice to transform the world’s cities into climate-smart, equitable and sustainable homes for all'^10^.

## Beyond smart cities

The concurrent global trends of urbanisation and climate change will require very smart and innovative solutions. However, it will take a lot more than a smart cities agenda to provide a more sustainable urban future. As the WUF 9 declaration concluded, it will require collaboration between all levels of government and partnerships to tackle the scale of the change ahead^11^. As cities grow, so does their consumption of natural resources and their dependency on Earth’s natural systems to thrive and prosper. Red alert days for extreme urban pollution in mega cities, recently in Beijing and Delhi signal a complex relationship between mega cities and regions^12^. For example, while urban pollution can be urban generated, it can also be the product of fires from logging in the region to provide resources for consumption by the growing city (e.g., soybean production in the Amazon, palm oil in south-east Asia and smallholder farming in central Africa) (see page 28 in ref. ^7^).

In the lead up to the inaugural IPCC Conference on Cities, six research priorities were identified for cities and climate change including the need for better urban data and a global network of ‘urban observatories’^13^. I could add an important seventh research priority being ‘urban governance’ including a better understanding of the multiple ways the developing science of cities can be incorporated into the planning, design and management of cities to activate the systems approach to the urban future. An example of such collaboration has been the series of reports and advice by the New York Panel on Climate Change bringing together the best scientific and planning expertise from the national to the local level on coastal flood risk, rising temperatures and rainfall to better prepare New York City for the impacts of climate change^14^.

## A sustainable urban agenda

Our urban future within the twenty-first century and beyond will inevitably impact the role and function of cities. As cities grow they will become stronger and more independent and autonomous economically as a result of their wealth. However, cities will remain, in my view, part of a complex set of environmental and social systems and as a result will continue to be influenced by the actions of higher levels of government. While cities can undertake significant local action on urban sustainability such as the 100% city renewable energy alliance^15^, they will continue to rely on national governments for investment in critical infrastructure including defence, energy, water supply, communications and rapid transit.

Where national strategies are aligned to local action, a great deal more can be achieved, as demonstrated by the removal of the major inner freeway in Seoul to restore life to the river Cheonggyecheon, which has resulted in environmental and community benefits and created one of Korea’s most popular tourist destinations, bringing national economic return^16^.

While cities grow economically more powerful, collaboration and partnerships will remain central to achieving green urban growth and the transformation required for a more sustainable future. So too will cooperation between nation states increase as urban city-regions spread across national borders.

Based in part on a recent series of interviews of urban leaders throughout the world, I put forward seven sustainable pathways for cities and regions in the future—(i) Planning within planetary boundaries, (ii) Long-term vision with targets, (iii) Adaptive integrated planning, (iv) National sustainable development strategies, (v) Net zero carbon precincts, (vi) Innovative platforms for collaboration and evaluation and (vii) Green growth, i.e., planning. Importantly, all seven steps require effective coordination at all levels of government for successful implementation (see page 149 in ref. ^7^). Again, the key message emerging from the interviews was the importance of a shared vision by all levels of government for our cities with clear targets for sustainability over both the immediate and longer term.

In conclusion, cities will become economically stronger and nation states will need to develop a more mature partnership with cities as they become integral to national performance, the health and well-being of citizens and global environmental outcomes. The performance of mega urban regions in relation to action on climate change and the environment more broadly (water, energy, air and land) will be critical to meeting the Paris Agreement targets and the United Nations Sustainable Development Goals. However, cities are an urban system within wider systems including Earth systems (water, energy, carbon, biodiversity and so on), social migration, global capital and more. In my view, we are in fact more connected than ever before. While cities will increasingly do many things very well at the urban level and through city networks, collectively we will develop a much more sustainable urban future when we are working together from the local to the global scale.
